# The relationship between mothers' maladaptive schemas and sleep problems in 12‐to‐36‐month‐old children: The role of attachment and sleep behaviors

**DOI:** 10.1002/imhj.70078

**Published:** 2026-03-23

**Authors:** Nursah Yilmaz, Ezgi Sen Yilmaz, Pinar Algedik

**Affiliations:** ^1^ Department of Psychology Private Practice Istanbul Turkey; ^2^ Department of Psychology Rumeli University Istanbul Turkey; ^3^ Department of Psychiatry Halic University Istanbul Turkey

**Keywords:** attachment behavior, cognition, early maladaptive schemas, mother‐child relations, parental, pediatric, sleep disorders

## Abstract

Sleep problems in early childhood are common and may be shaped by maternal psychological factors. This study, performed in Turkey, explored the association between mothers' early maladaptive schemas (EMS) and sleep difficulties in children aged 12–36 months. Mothers’ early maladaptive schemas were assessed using the Young Schema Questionnaire–Short Form 3 (YSQ‐S3), and sleep difficulties in children were defined according to BISQ‐derived clinical criteria. The sample consisted of 58.8% boys and 41.2% girls. Among 153 mothers, those whose children had sleep problems exhibited lower levels of “enmeshment/dependence” and “punitiveness” schemas. In contrast, higher maternal “failure to achieve” scores were associated with an increased likelihood of child sleep problems, while elevated “enmeshment/dependence” scores and receiving occasional support from a partner or family served as protective factors. Notably, maternal age over 30 emerged as a strong risk factor, whereas children aged 25–36 months demonstrated fewer sleep problems compared to younger counterparts. These findings point to possible associations between maternal schematic tendencies and child sleep characteristics across developmental contexts. These preliminary observations may highlight areas for further research on caregiver–child processes relevant to early childhood sleep.

## 1 INTRODUCTION

**Key findings**

1. “Mothers” “failure to achieve” schema significantly increases the risk of sleep problems in children aged 12‐to‐36 months, suggesting that maternal perceptions of incompetence may negatively affect children's sleep transition processes.
2. Higher “enmeshment/dependence” schema scores in mothers demonstrate a protective effect against children's sleep problems, potentially by enhancing emotional responsiveness that promotes secure attachment during this developmentally sensitive period.
3. Maternal age over 30 and the pattern of receiving occasional rather than constant support from family or spouse emerged as significant determinants of sleep problems in young children, highlighting the importance of age‐appropriate parenting approaches and flexible support systems.

**Diversity and anti‐racist scholarship**

Our study included diverse socioeconomic backgrounds across multiple regions of Turkey, acknowledging the cultural context of sleep practices within Turkish families. We intentionally used culturally sensitive measures validated for Turkish populations and avoided pathologizing cultural sleep practices that differ from Western norms. While our sample was relatively homogeneous in terms of cultural background, we recognized the limitations this presents and have interpreted our findings within the specific cultural context of Turkish parenting practices, emphasizing the importance of cultural sensitivity when developing sleep interventions for diverse populations.

**Statement of relevance**

This research advances understanding of how mothers' early maladaptive schemas influence children's sleep patterns, providing crucial insights for early intervention approaches. By demonstrating that specific maternal schemas can function as either risk or protective factors, our findings offer clinically relevant guidance for developing targeted sleep interventions that address parental cognitive structures alongside behavioral techniques, thus enhancing therapeutic effectiveness for infant and early childhood sleep problems.

Sleep problems are common in infants and toddlers (Lux et al., 2023). Rates of sleep problems, which occur in approximately one in five infants and toddlers, vary by age, how sleep problems are defined, and the methods used to assess sleep problems (Dias et al., 2018). While sleep problems in most children improve over time in the first 3 years of life, in some, they continue or even worsen. Sleep problems, especially when prolonged, can negatively affect the healthy psychophysiological development of children (Schmid et al., 2010). It has been reported that children with sleep problems have a high risk of experiencing emotional, behavioral, and cognitive difficulties (Nelson et al., 2015; Whalen et al., 2017). Therefore, it is crucial to identify the factors that may contribute to sleep problems in infants and toddlers and determine appropriate intervention methods to eliminate these problems. These factors include environmental factors such as light, noise, and temperature; room and bed conditions; the child's developmental level, including separation anxiety; baby‐specific factors such as medical conditions, for example, respiratory diseases such as asthma, gastroesophageal reflux, and parent‐related factors such as the way the parent interacts with the baby (Sadeh et al., 2010; Reid et al., 2009; Wei et al., 2022; Blum & Carey, 1996). Although core sleep–wake rhythms begin to emerge during early infancy (Sadeh et al., 2009), the period between 12 and 36 months is characterized by the further consolidation of sleep patterns and heightened sensitivity to environmental routines and parental caregiving behaviors (Mindell et al., 2015; Williamson et al., 2019). During this developmental window, factors such as separation anxiety, rapid motor and cognitive maturation, and parents’ strategies for managing night awakenings can play a decisive role in the persistence or resolution of sleep difficulties (Dias et al., 2018; Sadeh, 2005). Therefore, this age range represents a conceptually meaningful period for examining how parental cognitive characteristics may shape young children's sleep regulation. Both cross‐sectional (Graham et al., 2018) and longitudinal studies (Madigan et al., 2018) have shown that the mother's mental health, in particular, can negatively affect the mother‐child relationship, thereby negatively impacting the child's development and sleep health. In addition to these findings, previous research has shown that maternal mental health, particularly depressive symptoms during pregnancy and the postnatal period, may adversely influence infants’ sleep regulation (Orton & Bilgin, 2024). Furthermore, preterm birth has been linked to disruptions in neurodevelopmental pathways and sleep–wake organization, increasing vulnerability to early childhood sleep problems (Bilgin & Wolke, 2016, 2017). Among the various theoretical frameworks that explain how parental mental health influences the parent–child relationship, schema theory has gained increasing attention because early maladaptive schemas may shape parenting behaviors and, in turn, affect children's emotional and behavioral regulation. This makes schema theory particularly relevant to the aims of the present study.

Healthy sleep during early childhood is closely linked to environmental and behavioral sleep practices. Prior research suggests that independent sleep onset, falling asleep in one's own bed, and engaging in predictable calming routines (e.g., reading) support children's self‐soothing abilities and more consolidated nighttime sleep (Mindell et al., 2010, 2015). In contrast, behaviors such as bed‐sharing, being rocked or breastfed to sleep, and exposure to screens near bedtime have been associated with greater sleep fragmentation and difficulties in developing consistent sleep–wake patterns (Moon et al., 2016). These well‐established behavioral characteristics form the conceptual basis for the positive and negative sleep behavior categories used in the present study.

In the present study, we examined two distinct but complementary aspects of early childhood sleep. Sleep behaviors, including sleeping location, sleep onset method and pre‐sleep routines, were coded as positive or negative because previous research demonstrates that behavioral and environmental sleep practices play a central role in shaping sleep–wake regulation (Mindell & Williamson, 2018). Consistent bedtime routines, independent sleep onset and sleeping in a designated sleep space have been associated with longer nighttime sleep duration and more favorable sleep outcomes (Staples et al., 2015; Mindell & Lee, 2015). Evidence also indicates a dose‐dependent association between the consistency of bedtime routines and sleep outcomes (Mindell et al., 2015). In contrast, sleep problems refer to clinically meaningful indicators of sleep disturbance, defined according to validated BISQ thresholds and the diagnostic criteria outlined in the ICSD‐3 (Sadeh et al., 2009; Sateia, 2014). These two constructs serve different purposes: sleep behaviors reflect caregiving practices that support the development of healthy sleep patterns, whereas sleep problems represent observable disruptions in the child's sleep. Evaluating both dimensions together provides a more comprehensive understanding of how maternal schemas may be linked to caregiving practices and children's sleep outcomes.

Turkey‐specific contextual factors may also shape early childhood sleep patterns. Co‐sleeping, frequent nighttime caregiving, and culturally embedded soothing practices are commonly observed among Turkish families. Studies involving Turkish infants and young children suggest that sleep difficulties, including bedtime resistance and irregular sleep routines, may be relatively common in this context (Yalçıntaş‐Sezgin & Ulus, 2019; Cırdı et al., 2022). Evidence further indicates that sleeping arrangements, such as room sharing and bed sharing, may influence feeding and nighttime regulation among Turkish infants (Öztürk et al., 2023). Despite these findings, no studies have examined how maternal cognitive and emotional characteristics, including early maladaptive schemas, relate to sleep patterns in Turkish infants and toddlers. Addressing this gap may help elucidate culturally relevant mechanisms shaping early childhood sleep behaviors.

Early maladaptive schemas (EMS) refer to enduring cognitive and emotional patterns that are shaped by early developmental experiences. Prior research suggests that adverse childhood conditions, including various forms of neglect and maltreatment, may be associated with specific schema domains (Young et al., 2006; Sójta & Strzelecki, 2023). For example, emotional neglect has been linked to emotional deprivation and abandonment/instability schemas, while emotional, physical, or sexual abuse has been associated with schemas such as mistrust/abuse, defectiveness/shame, social isolation/alienation, and vulnerability to harm (Young et al., 2006; Sójta & Strzelecki, 2023). Such experiences may contribute to difficulties in emotion regulation and influence later caregiving tendencies. EMS have also been associated with parenting attitudes and mother–child interaction patterns, suggesting a possible pathway through which early experiences may shape intergenerational dynamics (Gibson & Francis, 2019; Sójta & Strzelecki, 2023; Young et al., 2006). Furthermore, EMS have been linked to poorer maternal well‐being, and higher schema levels have been associated with lower perceived parenting competence and reduced maternal self‐efficacy, both of which may affect the quality of mother–child interaction (Miklósi et al., 2017;Bellocchio et al., 2025).

Such negative perceptions of limited parenting competence may contribute to difficulties in the mother–infant relationship and challenges in meeting the infant's developmental needs, which can in turn affect early attachment (Nordahl et al., 2019). Consistent with this, a study from Norway involving 165 pregnant women reported significant negative associations between all EMS dimensions and mother–infant attachment scores (Nordahl et al., 2019).

Research suggests that sleep difficulties may be observed more frequently in infants with insecure attachment patterns, particularly in the form of frequent night awakenings and difficulties initiating sleep (Simard et al., 2013). Maternal EMS may also play a role in shaping caregiving behaviors that influence infant sleep. For instance, certain schemas could contribute to either under‐responsiveness or over‐involvement during nighttime caregiving, which may affect the infant's ability to develop consistent sleep–wake patterns. Prior studies have shown that parents’ dysfunctional cognitions about sleep, including difficulties with setting limits, are associated with poorer child sleep outcomes(Sadeh et al., 2007), and that parental beliefs may indirectly influence children's own sleep‐related cognitions (Ng et al., 2013). Overall, maternal maladaptive schemas may interact with caregiving behaviors in ways that influence both attachment‐related processes and sleep patterns during early development (Nordahl et al., 2019).

The present study was conducted during the COVID‐19 pandemic, a period in which restrictions on face‐to‐face contact substantially altered family routines and caregiving patterns. These circumstances may have influenced both infant sleep behaviors and maternal psychological well‐being, making the pandemic context an important factor when interpreting sleep‐related outcomes. Therefore, the developmental and relational processes examined in this study should be understood within the unique caregiving conditions created by COVID‐19 restrictions. In view of the foregoing, the objective of this study is to investigate the relationships between maternal EMS, mother–infant attachment, and sleep routines, as well as the associations between specific EMS dimensions and sleep problems in children aged 12–36 months.

Maternal early maladaptive schemas may influence processes such as interpreting infant cues, regulating emotional responses, and maintaining consistency in caregiving. Difficulties in these areas may reflect on the quality of mother–infant interaction and the development of the attachment relationship. Since attachment is also considered relevant to infants’ regulatory capacities, including sleep–wake organization, treating mother–infant attachment as a potential mediator between maternal schemas and child sleep characteristics appears conceptually meaningful.

Several contextual and caregiving‐related factors may influence how maternal early maladaptive schemas are reflected in daily parenting practices. For instance, maternal age may be associated with differences in emotional maturity, caregiving experience, and the ability to manage stress, which could shape how schema‐driven tendencies manifest in interactions with the child. Similarly, the consistency of sleep routines and the presence of spousal or family support may affect the extent to which mothers can regulate caregiving behaviors and manage challenges related to child sleep. Therefore, these characteristics may function as moderators that alter the strength or direction of the association between maternal schemas and children's sleep outcomes.

These aims were evaluated through the following hypotheses:

1. There is a significant relationship between maternal EMS levels and sleep problems in children aged 12–36 months.
2. Higher levels of schemas related to disconnection/rejection and impaired autonomy/performance, particularly “failure to achieve” and “emotional deprivation,” may be associated with an increased likelihood of sleep problems in children.
3. Mother–infant attachment may mediate the relationship between maternal EMS and children's sleep problems.
4. The quality of sleep routines and demographic characteristics, such as maternal age and spousal or family support, may moderate the relationship between maternal schemas and children's sleep problems.

## 2 MATERIALS AND METHODS

### 2.1 Study design

This cross‐sectional correlational study was conducted online due to restrictions imposed by the coronavirus disease 2019 (COVID‐19) pandemic between June and October 2020. The study protocol was approved by the university's ethics committee (Ethics Committee Number: 2020/03, Approval Date: 20.05.2020, Approval Number: 69396709‐050.01.04). The study's objective, scope, voluntary participation, and confidentiality principles were explained to the participants in detail, and their informed consent was obtained.

### 2.2 Population and sample

The study population consisted of mothers in Turkey who had at least one child aged 12–36 months and were able to understand the study procedures and complete the online form. Mothers who had more than one child within the 12–36‐month age range were excluded to ensure that each participating mother reported on a single target child. In addition, mothers with major depressive disorder, bipolar disorder, psychotic disorders, moderate‐to‐severe anxiety disorders, post‐traumatic stress disorder, or personality disorders requiring treatment, as well as children with neurodevelopmental disorders, behavioral disorders, chronic medical conditions, or any psychiatric diagnosis, were excluded. All parents provided written declarations confirming that neither they nor their children had any past or current psychiatric diagnoses. Because structured clinical evaluations could not be conducted during the COVID‐19 pandemic, exclusion criteria were assessed through parental self‐report, which may involve the possibility of undeclared or subthreshold conditions; this limitation was acknowledged when interpreting the findings.

No a priori power analysis could be conducted due to the constraints of online data collection during the COVID‐19 period. Therefore, the sample size was determined pragmatically and was guided by sample sizes commonly used in comparable studies in the literature. In total, the study sample consisted of 153 mothers of children aged 12–36 months who volunteered to participate.

### 2.3 Data collection

Mandatory restrictions brought about by the pandemic conditions resulted in the data collection process being designed and implemented online to ensure the participants' ease of access and security. Similarly, the inability to conduct structured diagnostic interviews or face‐to‐face evaluations due to the said mandatory restrictions necessitated psychiatric assessments to be based on the statements of the parents. Participants completed the online survey employed to collect study data in an average of 20–30 min. To prevent duplicate participation, participants' IP addresses were checked, and a unique identifier code was assigned to each participant. Possible duplicate entries were detected by checking for inconsistencies in demographic data, and the relevant data were removed from the data set.

### 2.4 Demographic and clinical data form

Demographic variables included maternal age, education, marital status, occupational and income status, and child‐related characteristics such as age, sex, birth order, siblings, and household composition. Clinical variables included child medical conditions, daycare attendance, caregiving support, and sleep‐related characteristics (sleep location, sleep onset method, nocturnal awakenings, and sleep routines).Sleep behaviors including sleeping location, sleep onset method and pre sleep routines were coded as positive or negative based on established pediatric sleep literature, and the operational definitions of these categories are presented in Table 1.

**TABLE 1:** Sleep behavior coding categories.

Categories	Positive coding example	Negative coding example
**Sleep location**	In own room, with sibling	In parents' bed, in living room
**Sleep onset method**	Independently, in own bed	While being held, breastfeeding, in front of television
**Pre‐sleep routine**	Reading books, calming routine	Screen exposure, physical activity

*Note*: Sleep behavior categories were coded as positive/negative in accordance with the literature on pediatric sleep medicine and developmental psychology principles.

### 2.5 Brief infant sleep questionnaire (BISQ)

BISQ, developed by Sadeh in 2004, expanded in 2009 and adapted to Turkish by Taşdemir and Bayık Temel in 2011, was used to evaluate the sleep patterns of infants and toddlers (Hemşire Fatma TAŞDEMİR et al., 2015; Sadeh et al., 2009). BISQ assesses seven basic parameters: sleep onset time, time taken to fall asleep, frequency of nocturnal awakenings, time spent awake at night, nighttime and daytime sleep duration, and total sleep duration. Total sleep duration is calculated by subtracting the time spent awake at night from the sum of nighttime and daytime sleep duration. The Cronbach alpha internal consistency coefficient of the BISQ is 0.90.

We constructed the sleep problems variable based on three parameters using BISQ data: (1) waking up ≥3 times at night, (2) spending > 60 min awake at night, or (3) time taken to fall asleep > 30 min. If at least one of these criteria was met, the relevant infant/toddler was coded as having “sleep problems”. These cut‐off points were created in line with Sadeh et al.’s recommendations (2007) and pediatric sleep disorders diagnostic criteria included in the International Classification of Sleep Disorders‐Third Edition (ICSD‐3) (Sadeh et al., 2007; Sateia, 2014). The ≥3 nocturnal awakenings threshold is widely accepted as a clinically meaningful indicator of disrupted nighttime regulation in toddlers and is consistent with developmental expectations for this age group, in whom 0–1 awakening are typically normative. In our sample, 86.4% of children classified as having sleep problems and 16.8% of those without sleep problems met this criterion, indicating that the threshold appropriately distinguished clinically meaningful variation in sleep.

### 2.6 Young schema questionnaire short form 3 (YSQ‐S3)

YSQ‐S3, developed by Jeffrey Young, was used to assess mothers’ EMS (Young et al., 2017). YSQ‐S3 includes a total of 90 items in 18 subscales, including emotional deprivation, failure to achieve, social isolation/alienation, approval seeking/recognition seeking, self‐sacrifice, and so forth, with five items in each subscale. Each item is scored on a 6‐point Likert‐type scale. Hence, the score of each subscale varies between 5 and 30. The adaptation of YSQ‐S3into Turkish and its validity and reliability studies were carried out by Soygüt, Karaosmanoğlu, and Çakır (Soygüt et al., 2009). Cronbach alpha internal consistency coefficients of YSQ‐S3 ranged between 0.53 and 0.81, while its test‐retest reliability was calculated as 0.66–0.82.

### 2.7 Mother‐to‐infant bonding scale (MIBS)

Eight‐item MIBS, developed by Taylor et al. to quickly assess the mother's emotional bond with her baby, was used to assess the mother‐child relationship (Taylor et al., 2005). Each item is scored on a 4‐ point Likert‐type scale. While the 1st, 4th, and 6th items of the MIBS include positive emotion expressions, the remaining items contain negative emotion expressions and are reverse scored. MIBS was adapted into Turkish by Karakulak Aydemir and Alparslan (Karakulak & Alparslan, 2016). Cronbach alpha internal consistency coefficients of MIBS ranged between 0.68‐0.70.

### 2.8 Statistical analysis

Statistical analyses were conducted using Jamovi 2.3.28, JASP 0.19.2, and R 4.4.2. Normality was examined with Kolmogorov–Smirnov and Shapiro–Wilk tests, and non‐parametric tests were used due to the non‐normal distribution of the data. Categorical variables were compared using Pearson's Chi‐square test, Fisher's exact test, or the Fisher–Freeman–Halton test, depending on expected cell counts. Numerical variables were compared using the Mann–Whitney *U* test.

Factors associated with sleep problems were examined using Firth's penalized logistic regression. Analyses were performed with the *logistf* package in R, and the *dplyr* and *forcats* packages were used for data handling and factor coding. Variables with *p* < 0.10 in univariate analyses were entered into the multivariate model. Although sleep behavior variables (i.e., sleeping location, sleep onset method, and pre‐sleep routines) were conceptually relevant, they were not included in the regression analysis because they did not demonstrate statistically significant group differences in univariate comparisons (*p* > 0.05), and thus did not meet the inclusion criterion. The backward elimination method was applied for model simplification, and model fit was evaluated using likelihood ratio tests and penalized pseudo‐*R* ^2^ values. Results were reported as odds ratios (OR) with 95% confidence intervals (CI).

Given the exploratory nature of the study and the use of multivariate Firth logistic regression, no additional multiple‐comparison correction was applied to univariate tests. This approach is recommended to control Type I error without inflating Type II error in small‐sample settings.

## 3 RESULTS

### 3.1 Sociodemographic characteristics of parents

In the total sample, 14.4% of children (22 out of 153) met the criteria for sleep problems. The distribution of the parents’ sociodemographic characteristics by the groups of children with and without sleep problems is given in Table 2. Accordingly, significant demographic differences were found between the parents of children with and without sleep problems (*p *≤ 0.05). In terms of mothers’ age, it was found that 95.5% and 63.4% of the mothers of children with and without sleep problems, respectively, were in the 30–40 age range (*p* = **0.012**). In terms of fathers’ profession, it was found that sleep problems were significantly more common in children whose fathers were teachers (18.2% vs. 3.1%) and healthcare workers (22.7% vs. 12.2%) (*p* = **0.038**). On the other hand, no significant difference was found between the children with and without sleep problems in terms of the mother's profession, parents' education level, and marital status (*p* > 0.05).

**TABLE 2:** Comparison of sociodemographic characteristics of parents of children with and without sleep problems.

Variables	Overall (*n* = 153)	Sleep problem present (*n* = 22, 14.4%)	Sleep problem absent (*n* = 131, 85.6%)	*p* value
**Child's gender**				
Female	63 (41.2)	12 (54.5)	51 (38.9)	0.253
Male	90 (58.8)	10 (45.5)	80 (61.1)	
**Maternal age** *(years)*				
20–30	35 (22.9)	1 (4.5)	34 (26.0)	**0.012**
30–40	104 (68.0)	21 (95.5)	83 (63.4)	
40–50	14 (9.2)	0 (0.0)	14 (10.7)	
**Paternal age** *(years)*				
20–30	8 (5.2)	0 (0.0)	8 (6.1)	0.236
30–40	119 (77.8)	21 (95.5)	98 (74.8)	
40–60	26 (17)	1 (4.5)	25 (19.1)	
**Maternal education**				
High school	22 (14.4)	3 (13.6)	19 (14.5)	0.999
University	131 (85.6)	19 (86.4)	112 (85.5)	
**Paternal education**				
No formal education	1 (0.7)	0 (0.0)	1 (0.8)	0.505
Primary school	1 (0.7)	0 (0.0)	1 (0.8)	
Middle school	7 (4.6)	1 (4.5)	6 (4.6)	
High school	22 (14.4)	1 (4.5)	21 (16.0)	
University	122 (79.7)	20 (90.9)	102 (77.9)	
**Maternal occupation**				
Homemaker	27 (17.6)	7 (31.8)	20 (15.3)	0.095
Healthcare professional	31 (20.3)	6 (27.3)	25 (19.1)	
Teacher	29 (19.0)	5 (22.7)	24 (18.3)	
Engineer	10 (6.5)	0 (0.0)	10 (7.6)	
Other	56 (36.6)	4 (18.2)	52 (39.7)	
**Paternal occupation**				
Laborer	3 (2.0)	1 (4.5)	2 (1.5)	**0.038**
Self‐employed	13 (8.5)	1 (4.5)	12 (9.2)	
Civil servant	7 (4.6)	1 (4.5)	6 (4.6)	
Healthcare professional	21 (13.7)	5 (22.7)	16 (12.2)	
Teacher	8 (5.2)	4 (18.2)	4 (3.1)	
Engineer	27 (17.6)	2 (9.1)	25 (19.1)	
Other	74 (48.4)	8 (36.4)	66 (50.4)	
**Marital status**				
Together	152 (99.3)	22 (100.0)	130 (99.2)	0.999
Other	1 (0.7)	0 (0.0)	1 (0.8)	

*Note*: Data are presented as *n* (%). Categorical variables were compared using Pearson's Chi‐square test (for 2×2 tables with expected values >5), Fisher‐Freeman‐Halton exact test (for R×C tables with expected values <5), and Fisher's exact test (for 2×2 tables with expected values <5). Bold p‐values indicate statistical significance (*p* ≤ 0.05)

### 3.2 Child sleep parameters and behavioral characteristics

The distribution of children's demographic characteristics, sleep parameters, and behavioral characteristics by the groups of children with and without sleep problems is given in Table 3. Accordingly, the number of children with sleep problems in the younger age groups was significantly higher than those without sleep problems (*p* = **0.033**). Sleep problems were detected in only **36.4%** of the children in the 25‐to‐36 month age group, compared to **45.5%** in the 12‐to‐18 month age group. As for sleep parameters, the frequency of nocturnal awakenings was significantly higher in children with sleep problems than in children without sleep problems (*p* = **<** **0.001**). Accordingly, compared to **86.4%** of children with sleep problems, only **16.8%** of children without sleep problems were waking up three or more times during the night. On the other hand, no significant difference was found between the children with and without sleep problems in terms of behavioral characteristics such as screen use and crying spells (*p* > 0.05).

**TABLE 3:** Comparison of sleep parameters and demographic characteristics of children with and without sleep problems.

Variables	Overall (*n* = 153)	Sleep Problem Present (*n* = 22)	Sleep Problem Absent (*n* = 131)	p‐value
**Demographic and clinical characteristics**				
**Age group**				
12–18 months	37 (24.2)	10 (45.5)	27 (20.6)	
19–24 months	26 (17.0)	4 (18.2)	22 (16.8)	**0.033**
25–36 months	90 (58.8)	8 (36.4)	82 (62.6)	
**Pregnancy**				
Planned	130 (85.0)	19 (86.4)	111 (84.7)	
Unplanned	23 (15.0)	3 (13.6)	20 (15.3)	0.999
**Child has own room** *(yes)*	116 (75.8)	17 (77.3)	99 (75.6)	0.999
**Pacifier use**				
Yes	31 (20.3)	3 (13.6)	28 (21.4)	
No	100 (65.4)	17 (77.3)	83 (63.4)	0.542
Discontinued	22 (14.4)	2 (9.1)	20 (15.3)	
**Sleep Parameters**				
**Frequency of nocturnal awakenings** *(times)*				
0–2 times	112 (73.2)	3 (13.6)	109 (83.2)	
3–5 times	24 (15.7)	9 (40.9)	15 (11.5)	**<0.001**
6 or more	17 (11.1)	10 (45.5)	7 (5.3)	
**Total sleep duration**				
Less than 9 h	2 (1.3)	0 (0.0)	2 (1.5)	
9 h or more	151 (98.7)	22 (100.0)	129 (98.5)	0.999
**Screen exposure and behavioral characteristics**				
**Daily screen exposure**				
0–1 h	85 (55.6)	13 (59.1)	72 (55.0)	
1–3 h	48 (31.4)	8 (36.4)	40 (30.5)	
3–5 h	14 (9.2)	0 (0.0)	14 (10.7)	0.425
5–7 h	4 (2.6)	1 (4.5)	3 (2.3)	
More than 9 h	2 (1.3)	0 (0.0)	2 (1.5)	
**Pre‐sleep screen exposure** *(yes)*	47 (30.7)	4 (18.2)	43 (32.8)	0.259
**Crying spells**	74 (48.4)	7 (31.8)	67 (51.1)	0.148
When hungry	21 (13.7)	3 (13.6)	18 (13.7)	0.999
When refusing to eat	10 (6.5)	0 (0.0)	10 (7.6)	0.359
When sleepy	39 (25.5)	3 (13.6)	36 (27.5)	0.265
Upon awakening	10 (6.5)	1 (4.5)	9 (6.9)	0.999
When demands are not met	53 (34.6)	4 (18.2)	49 (37.4)	0.131
Other	2 (1.3)	0 (0.0)	2 (1.5)	0.999
**Nocturnal crying spells** *(yes)*	32 (20.9)	6 (27.3)	26 (19.8)	0.408

*Note*: Data are presented as *n (%)*, with percentages shown in parentheses. Categorical variables were compared using Pearson's Chi‐square test (for tables with adequate expected values), Fisher‐Freeman‐Halton exact test (for multi‐cell tables with inadequate expected values), and Fisher's exact test (for 2 × 2 tables with inadequate expected values). Bold *p*‐values indicate statistical significance (*p* ≤ 0.05).

### 3.3 Sleep routines and family support

The distribution of children's sleep routines, sleep‐related behaviors, and whether the mother receives support from the family or spouse in child care by the groups of children with and without sleep problems is given in Table 4. In terms of children's sleep environment and sleep transition characteristics, no significant differences were found between the children with and without sleep problems in terms of where they slept, where they lay down, and how they fell asleep. On the other hand, regarding the duration of uninterrupted sleep at night, significant differences were found between the children with and without sleep problems (*p* = **0.002**). Accordingly, **77.3%** of children with sleep problems were able to sleep uninterruptedly for a maximum of 6 h, while **64.6%** of children without sleep problems were able to sleep uninterruptedly for 7 h or more. In addition, although no significant difference was found between the children with and without sleep problems in terms of whether the mother was receiving support from family or spouse in child care (*p* = 0.202), it was found that mothers of children with sleep problems were receiving less support from family or spouse in child care than mothers of children without sleep problems.

**TABLE 4:** Comparison of sleep routines, behaviors, and family support between children with and without sleep problems.

Variables	Overall (*n* = 153)	Sleep problem present (*n* = 22)	Sleep problem absent	*p* value
**Sleep environment and routines**				
**Where does your baby usually sleep?**				
Negative	80	10	70	0.644
	(52.3)	(45.5)	(53.4)	
Positive	73	12	61	
	(47.7)	(54.5)	(46.6)	
**Where does your baby usually lie down?**				
Negative	44	6 (27.3)	38	0.999
	(28.8)	16	(29.0)	
Positive	109	16	93	
	(71.2)	(72.7)	(71.0)	
**Pre‐sleep activities frequently applied to baby**	146	22	124	0.594
**POSITIVE**	(95.4)	(100.0)	(94.7)	
**Pre‐sleep activities frequently applied to baby**	97	13	84	0.830
**NEGATIVE**	(63.4)	(59.1)	(64.1)	
**How does your baby usually fall asleep? POSITIVE**	7 (4.6)	1 (4.5)	6 (4.6)	0.999
**How does your baby usually fall asleep? NEGATIVE**	150	22	128	0.999
	(98.0)	(100.0)	(97.7)	
**Sleep onset duration**				
Less than 5 min	6 (3.9)	0 (0.0)	6 (4.6)	0.145
5–15 min	39	4 (18.2)	35
	(25.5)		(26.7)	
16–30 min	73	10	63	
	(47.7)	(45.5)	(48.1)	
31–60 min	29	5 (22.7)	24	
	(19.0)		(18.3)	
More than 1 h	6 (3.9)	3 (13.6)	3 (2.3)	
Night sleep characteristics and family support				
What do you do when your baby wakes up at night?	51	6 (27.3)	45	0.684
**POSITIVE**	(33.3)		(34.4)	
**What do you do when your baby wakes up at night?**	137	21	116	0.471
**NEGATIVE**	(89.5)	(95.5)	(88.5)	**0.002**
**Duration of uninterrupted sleep at night**				
1–3 h	16 (10.5)	6 (27.3)	10 (7.7)	0.202
4–6 h	47	11	36	
	(30.9)	(50.0)	(27.7)	
7–9 h	37	2 (9.1)	35	
	(24.3)		(26.9)	
10 h or more	52	3 (13.6)	49	
	(34.2)		(37.7)	
Does the mother receive support from family or spouse?				
Never	30	7 (31.8)	38	
	(19.6)		(29.0)	
Sometimes	78	7 (31.8)	23	
	(51.0)		(17.6)	
Usually	45	8 (36.4)	70	
	(29.4)	(53.4)	

*Note*: Data are presented as *n* (%). Categorical variables were compared using Pearson's Chi‐square test, Fisher's exact test, and Fisher‐Freeman‐Halton exact test based on appropriate cell counts. Bold *p*‐values indicate statistical significance (*p* ≤ 0.05)

### 3.4 Maternal early maladaptive schemas and mother–infant bonding

The distribution of mothers’ EMS structures assessed by YSQ‐S3 and mother‐baby attachment levels assessed by MIBS by the groups of children with and without sleep problems is given in Table 5. The median YSQ‐S3 enmeshment/dependence and punitiveness schema scores of mothers of children with sleep problems were significantly lower than those of mothers of children without sleep problems (10.5 vs. 13.0, *p* = **0.036** and 16.5 vs. 19.0, *p* = **0.023**). In terms of YSQ‐S3 overcontrol/emotional inhibition and unrelenting standards/hypercriticalness schema scores, nearly significant differences were found between mothers of children with sleep problems and mothers of children without sleep problems (*p* = 0.089 and *p* = 0.055, respectively). On the other hand, no significant difference was found between the children with and without sleep problems in terms of MIBS scores (*p* = 0.645).

**TABLE 5:** Comparison of early maladaptive schemas and mother‐infant bonding levels between mothers of children with and without sleep problems.

Variables	Overall (*n* = 153)	Sleep problem present (*n* = 22)	sleep problem absent (*n* = 131)	*p*‐value
**Young schema questionnaire**				
Emotional deprivation	7.0 [5.0–30.0]	5.5 [5.0–19.0]	7.0 [5.0–30.0]	0.176
Failure to achieve	10.0 [6.0–34.0]	10.0 [6.0–23.0]	10.0 [6.0–34.0]	0.857
Pessimism	10.0 [5.0–30.0]	10.0 [5.0–27.0]	10.0 [5.0–30.0]	0.952
Social isolation/insecurity	14.0 [7.0–39.0]	14.0 [7.0–19.0]	14.0 [7.0–39.0]	0.267
Emotional inhibition	9.0 [5.0–28.0]	7.0 [5.0–22.0]	10.0 [5.0–28.0]	0.089
Approval seeking	19.0 [6.0–36.0]	18.5 [7.0–30.0]	19.0 [6.0–36.0]	0.942
Enmeshment/dependence	12.0 [9.0–36.0]	10.5 [9.0–23.0]	13.0 [9.0–36.0]	**0.036**
Entitlement/insufficient self‐control	22.0 [7.0–39.0]	19.0 [8.0–32.0]	22.0 [7.0–39.0]	0.277
Self‐sacrifice	15.0 [5.0–30.0]	14.0 [5.0–27.0]	16.0 [5.0–30.0]	0.206
Abandonment	6.0 [5.0–20.0]	5.0 [5.0–15.0]	6.0 [5.0–20.0]	0.157
Punitiveness	19.0 [6.0–34.0]	16.5 [6.0–23.0]	19.0 [6.0–34.0]	**0.023**
Defectiveness	6.0 [6.0–24.0]	7.0 [6.0–20.0]	6.0 [6.0–24.0]	0.958
Vulnerability to harm	9.0 [5.0–23.0]	8.0 [5.0–16.0]	9.0 [5.0–23.0]	0.662
Unrelenting standards	7.0 [3.0–18.0]	5.5 [3.0–16.0]	7.0 [3.0–18.0]	0.055
**Mother‐Infant Bonding Scale (MIBS)**				
Total Score	22.0 [3.0–24.0]	22.0 [10.0–24.0]	22.0 [3.0–24.0]	0.645

*Note*: Data are presented as median [minimum–maximum]. Numerical variables were compared between groups using Mann–Whitney U test. Bold *p*‐values indicate statistical significance (*p* < 0.05).

### 3.5 Predictors of sleep problems

The results of Firth's logistic regression analysis performed to determine the factors that predict sleep problems are presented in detail in Table 6. Univariate analysis revealed that maternal age over 30 years (OR: 5.07, 95% CI: 1.23–37.70, *p* = **0.022**) and YSQ‐S3 enmeshment/dependency (OR: 0.92, 95% CI: 0.81‐0.99, *p* = **0.034**) and punitiveness (OR: 0.91, 95% CI: 0.84–0.98, *p* = **0.017**) schema scores were significantly associated with sleep problems. Multivariate analysis revealed that maternal age over 30 (OR: 6.40, 95% CI: 1.36–42.66, *p* = **0.015**) and high YSQ‐S3 failure to achieve schema score (OR: 1.11, 95% CI: 1.00–1.24, *p* = **0.049**) were risk factors for children having sleep problems, while the child being in the 25‐to‐36 month age group (OR: 0.39, 95% CI: 0.14–0.94, *p* = **0.037**), the mother sometimes receiving support from her spouse (OR: 0.21, 95% CI: 0.06–0.72, *p* = **0.013**) and highYSQ‐S3 enmeshment/dependence schema score (OR: 0.96, 95% CI: 0.86–0.97, *p* = **0.041**) were protective factors. The final model created as a result of the analysis explained approximately 25% of the variance (Penalized Pseudo‐*R* ^2^ = 0.25).

**TABLE 6:** Univariate and multivariate Firth's logistic regression analysis of factors predicting sleep problems.

Variables	Univariate analysis OR [95% CI]	*p*‐value	Multivariate analysis OR [95% CI]	*p*‐value
**Maternal age**				
*30–50 years versus 20–30 years*	5.07 [1.23–37.70]	**0.022**	6.40 [1.36–42.66]	**0.015**
**Child's Age**				
*25–36 months versus 12–18 months*	0.31 [0.12–0.80]	**0.016**	0.39 [0.14–0.94]	**0.037**
**Spousal support**				
*Sometimes receiving versus Never receiving*	0.38 [0.13–0.95]	0.084	0.21 [0.06–0.72]	**0.013**
*Usually receiving versus Never receiving*	0.61 [0.20–1.93]	0.395	0.50 [0.15–1.70]	0.262
**Early maladaptive schemas**				
Enmeshment/dependence	0.92 [0.81–0.99]	**0.034**	0.96 [0.86–0.97]	**0.041**
Punitiveness	0.91 [0.84–0.98]	**0.017**	0.92 [0.82–1.02]	0.118
Emotional deprivation	0.94 [0.83–1.03]	0.178	–	–
Failure to achieve	0.99 [0.91–1.07]	0.844	1.11 [1.00–1.24]	**0.049**
Emotional inhibition	0.92 [0.83–1.01]	0.094	1.00 [0.89–1.11]	0.975
Unrelenting standards	0.89 [0.76–1.01]	0.069	0.95 [0.80–1.11]	0.520
**Mother‐infant bonding scale**				
Total score	0.95 [0.86–1.06]	0.339	–	–

*Note*: Firth's logistic regression method was used. Variables with *p* < 0.10 in univariate analysis were included in the multivariate analysis. Model fit was evaluated with LR test = 21.75, *p* = 0.005 and Penalized Pseudo‐*R* ^2^ = 0.25. Bold *p*‐values indicate statistical significance (*p* ≤ 0.05).

Abbreviations: OR, odds ratio; CI, confidence interval.

## 4 DISCUSSION

This study investigated the relationships between mothers' EMS patterns and sleep problems observed in their 12‐to‐36‐month‐old children. Consequently, mothers' EMS schemas of “failure to achieve” and “enmeshment/dependence” were found to be significantly associated with sleep problems observed in their 12‐to‐36‐month‐old children. Similarly, one study reported that increased “failure to achieve” schema scores in mothers significantly increased the risk of sleep problems in their children (Sójta & Strzelecki, 2023). This finding suggests a possible influence of the mother's perceived parenting competence on her child's sleep transition process. Mothers who feel incompetent and unsuccessful are more likely to show overly intrusive or inconsistent reactions when their children fall asleep, which can negatively affect the child's emotional regulation and make the sleep process difficult (Young et al., 2006). These interventions may delay the child's ability to fall asleep on his/her own, preventing the development of sleep‐related autonomy. It is critical for children to develop independent sleep skills in terms of establishing biological rhythms and supporting self‐regulation capacity (St James‐Roberts et al., 2017).

We found that an increase in mothers' “enmeshment/dependence” schema scores significantly associated with a decrease in the risk of sleep problems in their children. This finding partially contradicts traditional expectations stated in the literature, as the “enmeshment/dependence” schema is often associated with overprotective parenting. It has been suggested that the “enmeshment/dependence” schema supports a parenting style that limits the child's age‐appropriate autonomy development, encourages overdependence, and inhibits the child's interaction with the outside world (Yap et al., 2014). Such a parenting style may cause delays in the child's psychosocial development and increased susceptibility to psychiatric disorders such as anxiety (Yap & Jorm, 2015).

The inverse relationship we detected between mothers' “enmeshment/dependence” schema scores and sleep problems in their children may gain additional meaning when interpreted from the perspective of attachment theory. According to attachment theory, the secure relationship that an infant establishes with his or her caregiver forms the basis for developing emotion regulation and self‐ regulatory behaviors, especially in the first years of life (Bowlby, 1982). Accordingly, mothers with high “enmeshment/dependence” schema scores might be more inclined to adopt a care style that is more sensitive to the child's emotional needs, prioritizes physical closeness, and responds to signals more quickly. In this way, the child's sense of security related to sleep could potentially be strengthened, paving the way for developing a healthier sleep pattern. As a matter of fact, it has been demonstrated through different measurement methods that infants who develop secure attachments have more regular sleep patterns (Perpétuo et al., 2021). Hence, the “enmeshment/dependence” schema may play a protective role in developmental processes such as sleep in certain contexts, such as early childhood, where it is directly reflected in emotional care behaviors. Therefore, it should be considered that this schema, the negative features of which are often emphasized in the literature, might exert varying effects on the child's sleep problems in the short term, depending on the child's age, the nature of the attachment process, and the characteristics of the care environment. In this respect, the current study aims to provide a critical perspective against rigid schema‐problem correlations in the field of EMS and draws attention to the necessity of context‐ based evaluations. From the perspective of clinical psychiatry, our findings highlight the possibility that sleep problems in early childhood are not merely a developmental problem but should also be addressed within the framework of pediatric sleep disorders. Specific sleep disorders, such as pediatric insomnia, parasomnias, or circadian rhythm disorders, could be influenced by the parents’ EMS structures and psychopathologies. It should be considered that increased anxiety levels, especially in mothers with increased “failure to achieve” schema, may predispose them to anxiety disorder symptoms or anxiety‐ based sleep disorders in their children. Similarly, the “enmeshment/dependence” schema in mothers might be linked to borderline personality organization or dependent personality traits, which may shape attachment patterns in the mother‐child relationship, affecting the child's sleep regulation.

We found that mothers being 30 or older was significantly associated with sleep problems in their children. This observation aligns with findings in the literature that late‐life motherhood may increase psychological burden and cause an increase in anxiety levels in particular (Molina‐García et al., 2019). Increased parental anxiety may lead to oversensitivity to the child's needs, leading to intrusive caregiving styles that prevent the child from developing self‐soothing skills. This association underscores the potential role of care giving attitudes that support the development of independence in the transition to sleep and raises the possibility that the high sensitivity that comes with older age could adversely affect this process (Nordahl et al., 2019).

Another notable finding of our study is that spouses’ occasional emotional and task‐sharing support to mothers was associated with a lower likelihood of sleep problems in their children. This suggests that a flexible and need‐sensitive support structure provided in times of need, rather than a fixed and continuous level of support, might be linked to fewer sleep problems in children. It has been reported that the mother's feeling of being supported reduces her anxiety level, which has a positive effect on her parenting skills and increases consistency in her interaction with her child (Nordahl et al., 2019). Receiving intermittent and needs‐based support in child care prevents the mother from feeling lonely and allows her to maintain her self‐sufficiency.

On the other hand, receiving constant support may not always yield positive results. Mothers who constantly receive help might become passive in parenting processes over time and may experience decreased self‐efficacy and independent decision‐making skills. Receiving constant support may also lead to parental inconsistency, which could negatively influence the child's emotional safety. Additionally, a support process that interferes with the mother's area of responsibility may cause pressure and stress on the mother (Feeney & Collins, 2015). These dynamics may contribute to a psychosocial environment that can negatively affect the child's sleep patterns, even if the mother is provided with support. Therefore, not only the existence of family support systems but also their quality, timing, and compatibility with the mother's psychological appear to be important in the adaptation process of children to their biological rhythms (Sadeh et al., 2009). In other words, it is essential for child development that the level of support is not necessarily high but that it is provided in a flexible and sensitive manner.

Another finding of our study was that mother‐infant attachment levels, which we assessed using MIBS, did not show a statistically significant relationship with sleep problems in children. This unexpected result may be due to the assessment tool not adequately reflecting the qualitative dimensions of attachment or perhaps the effect of attachment on sleep is more pronounced during the first 12 months of life (Ng et al., 2013). It is also possible that variation in the children's responses to environmental stimuli with age and the introduction of different developmental factors that may mask the effect of attachment may have reduced the statistical significance of this relationship.

Our study aimed to contribute to the literature by exploring the potential associations between EMS and both the psychopathological characteristics of the mother and the biological rhythms of the child. In particular, the opposing effects of “failure to achieve” and “enmeshment/dependence” schemas on children's sleep patterns suggest that schemas may produce different results in a context‐sensitive manner. By focusing on early childhood, this study adds to the limited body of research examining schema theory within a neurodevelopmentally sensitive period, as most previous studies have concentrated on older age groups. Our findings **point to the importance of** addressing childhood sleep problems not only through behavioral habits but also through multidimensional factors, including parents’ life experiences, cognitive structures, and level of social support. For this reason, intervention plans could benefit from being structured to take into account not only the child but also the parent's schematic tendencies and emotional functionality. Particularly in infancy, parental guidance programs based on the principle of “closeness with sensitivity” may support the development of a sense of security and healthy sleep habits in the child. It is important to consider that parents' cognitive schemas may have an indirect but strong impact on their children's self‐regulation skills and biological rhythms.

### 4.1 Limitations of the study

There were several limitations to this study. Firstly, considering that the research was conducted in 2020, the inability to conduct face‐to‐face clinical interviews with participants and structured diagnostic evaluations, as the research data had to be collected online due to the mandatory restrictions imposed by the conditions of the COVID‐19 pandemic, constituted a significant methodological limitation. Secondly, the relatively small number of children with sleep problems (*n* = 22) weakened the statistical power of subgroup analyses. Thirdly, carrying out psychopathology assessments of both mothers and children based solely on parental statements has led to the inability to control subthreshold symptoms or undiagnosed psychiatric conditions. In particular, not evaluating whether the mother has common psychopathologies such as depression and anxiety with objective criteria may have made it difficult to distinguish the relationship between schema levels and the underlying psychopathology. Similarly, the developmental or neuropsychiatric causes underlying sleep problems observed in children could not be differentiated due to the inability to perform face‐to‐face evaluations. Evaluation of children's sleep behaviors based only on parental statements and the inability to directly observe mother‐child interaction may have limited the objectivity of our assessments. The real‐life implications of the attachment quality and sleep routines reported by parents could not be assessed. It should also be noted that parental responses leading to sleep behaviors classified as “positive” and “negative” may have been influenced by cultural contexts and social desirability effects. Despite all these limitations, the high validity of the assessment tools used in the study, their compatibility with the international pediatric sleep literature, and the meticulous conduct of the data collection process despite the pandemic conditions are expected to support the internal consistency of the study. Another limitation is that important child‐related factors such as temperament, neurodevelopmental conditions, and sensory processing difficulties were not assessed, although these characteristics are known to influence sleep patterns in early childhood. The absence of these measurements may have contributed to variability in sleep outcomes and should be addressed in future studies.

Moreover, the cross‐sectional design of the study did not allow us to examine the temporal course of sleep problems, which are known to fluctuate over time in response to developmental changes and variations in the family environment. Given these limitations, the findings of this study should be interpreted with caution and regarded as exploratory. Longitudinal studies are needed to reveal the temporal changes in the effect of mothers' schematic tendencies on children's sleep patterns. In addition, future studies should further examine the interaction of EMS and different attachment styles using objective sleep assessment methods such as actigraphy and also include fathers’ schematic structuring in the modeling.

## 5 CONCLUSIONS

In conclusion, taking parents' schematic structures into account in evaluating childhood sleep problems may offer preliminary insights that could inform the planning of developmentally and clinically appropriate interventions.

## FUNDING INFORMATION

The authors received no financial support for this study.

## CONFLICT OF INTEREST STATEMENT

The authors declare that they have no conflict of interest.

## Data Availability

The data that support the findings of this study are available from the corresponding author upon reasonable request.
